# Molecular manipulations of miR398 increase rice grain yield under different conditions

**DOI:** 10.3389/fpls.2022.1037604

**Published:** 2022-11-07

**Authors:** Yuzhu Lu, Kena Yao, Zhiyun Gong, Yixin Zhang, Yunlong Meng, Qiaoquan Liu

**Affiliations:** ^1^ College of Bioscience and Biotechnology, Yangzhou University, Yangzhou, China; ^2^ Joint International Research Laboratory of Agriculture and Agri-Product Safety, the Ministry of Education of China, Yangzhou University, Yangzhou, Jiangsu, China; ^3^ Key Laboratory of Plant Functional Genomics of the Ministry of Education, College of Agriculture, Yangzhou University, Yangzhou, China

**Keywords:** miR398, Cu/Zn superoxide dismutases (CSDs), salty stress, short tandem target mimic (STTM), saline-alkali soil

## Abstract

Rice miR398 targets two stress-tolerant genes, *CSD1*-*2 (Cu/Zn Superoxide Dismutases1*-*2)* and *CCS* (copper chaperone of CSD), which usually boost plants’ tolerance by inhibiting growth. So, how to accurately regulate the activities of miR398 targets and thus make rice better able to adapt to different conditions has great significances in producing rice yields under the current circumstances of shrinking arable lands resulting from global urbanization and increasing salty soil caused by irrigation. Through controlling the expressions of miR398 in different levels, we found down-regulated expression of miR398 targets can promote growth under good growth conditions while up-regulated expressions of the targets can help rice tolerate salt. In this study, we over-expressed miR398 highly, moderately, and lowly, then three concomitantly inverse levels of its targets’ expression were obtained. Under normal growth conditions, the transgenic lines with low and moderate levels of over-expressions of miR398 could increase grain yields 14.5% and 7.3%, respectively, although no transgenic lines could survive well under salty conditions simulating real saline-alkali soil. Using short tandem target mimic (STTM) technology to silence miR398 highly, moderately, and lowly respectively, also three inverse levels of its targets’ expression were obtained. All three transgenic lines exhibited good agronomic performances under salt stress in inverse to their degrees of STTM, but their growth was inhibited differently under normal conditions. Altogether, we suggest that flexibly manipulating the expression of miR398 is an ideal strategy to help rice survive better and achieve optimized yields under specific conditions.

## Introduction

Around 20% of cultivated lands and nearly half of all irrigated lands are negatively affected by salt (Zhu, 2001; Deinlein et al., 2014; Liang et al., 2018). Salt accumulation in arable soils mainly stems from irrigation water containing trace amounts of sodium chloride (NaCl) or from seawater (Tester and Davenport, 2003). Plants undergo genetic changes as well as impaired metabolism and photosynthesis under salt stress (Mäser et al., 2002). The integrity of cellular membranes, activities of various enzymes, acquisition of nutrients, and function of photosynthetic apparatus are all prone to be harmed by the toxic effects of salt stress (Zhu, 2001). Crops experience huge yield losses caused by inhibited growth under salt stress. Thus, the formidable challenges are emerging urgently when such yield losses meet with tremendous demands for foods from the rapidly expanding population worldwide. In this context, how to genetically modify crops to achieve enhanced salt-tolerance may be a promising option to address these challenges. On the other hand, arable land is being lost at a fast speed due to the trend of global urbanization, therefore, how to increase rice yield on limited arable lands also has significance.

The mechanism of the damage originating from salt on plants is not entirely clear. An important clue might be attributed to reactive oxygen species (ROS) generated from salt stress, as ROS is thought to play dual roles in plant biology. They are required for many important signaling reactions, but are also toxic byproducts of aerobic metabolism (Mittler, 2017). Superoxide dismutase (SOD), the first line of defense of the plant antioxidant enzyme system, can remove excess superoxide anions in the cells (Liang et al., 2018). Over the past three decades, the genes encoding SODs have been identified from various plants (Tsang et al., 1991; Scandalios, 1993; Kim et al., 1996; Kliebenstein et al., 1998; Wang et al., 2016a; Wang et al., 2016b). In higher plants, SODs are classified into three groups according to the different metal cofactors: manganese SOD (Mn-SOD), iron SOD (Fe-SOD), and copper/zinc SOD (Cu/Zn-SOD, CSD) (Bowler et al., 1992). CSD1, CSD2, and CSD3 are located in the cytoplasm, chloroplasts, and peroxisomes, respectively, and their expressions are differently regulated (Kliebenstein et al, 1998). *CSD1* and *CSD2* are up-regulated under various stresses, such as ozone, UV-B, and high levels of light (Sharma and Davis, 1997; Kliebenstein et al., 1998). Although *SOD* genes are believed to be a class of stress-responsive genes, evidence from the over-expression of stress-tolerant components also suggests that there are likely relationships between stresses and growth regulation. Constitutive over-expression of several stress-related genes has been shown to cause slow growth of transgenic plants (Söderman et al., 1996; Liu et al., 1998; Zhang et al, 2017). These results suggested that stress-tolerant genes usually help plants get through predicaments at the price of inhibiting growth.

Both *Cu/Zn Superoxide Dismutases1* (*CSD1*) and *Cu/Zn Superoxide Dismutases2* (*CSD2*), along with *CCS* (copper chaperone of CSD1 and CSD2, and delivers copper to these two SODs) and *COX5b–1* in *Arabidopsis thaliana*, have been identified as the targets of the small non-coding RNA, miR398 (Bonnet et al., 2004; Jones-Rhoades and Bartel, 2004; Guan et al., 2013). Plant microRNAs serve as a class of sensitive regulators controlling their targets’ mRNA level due to a higher degree of complementarity (Rhoades et al., 2002; Pandey et al., 2019). The dynamic fluctuation of a microRNA’s expression usually results in corresponding varieties of multiple targets’ expressions in plants (Rhoades et al., 2002; Pandey et al., 2019). Relatively, a higher possibility to get a visible phenotype in a transgenic plant with ectopic expression of a microRNA easily occurs, owing to the regulation of that microRNA on multiple targets (Jones-Rhoades et al., 2006; Song et al., 2019). So far, many transgenic lines with evident phenotype by increasing or decreasing the expressions of plant microRNAs have been reported (Jones-Rhoades et al., 2006; Yu et al., 2017; Zhang et al., 2017; Song et al., 2019). As a famous stress-responsive star, miR398 and its targets of *CSD 1-2* and *CCS* have been proven to be involved in many environmental responses (Sunkar et al., 2006; Li et al., 2010; Lu et al., 2011; Javed et al, 2019; Siddiqui et al., 2019; Li et al., 2020; Sun et al., 2020).

Our previous works have provided evidence that rice miR398 responds to abiotic and biotic stresses through regulating the expression of its target genes, *OsCSD1* and *OsCSD2* (Lu et al., 2011). Since then, we also found that a relationship exists between the expressions of miR398 targets and yield in the following years of planting these transgenic lines. Here we report that both over-expressed and silenced miR398 at three different levels could have major effects on the yield of rice. By comparing the agronomic performances of these lines under normal conditions or mild salt stress simulating the real Saline-alkali land, we obtained the optimal options under given conditions. What we found here could provide an ideal strategy to help crops survive better under different conditions by engineering crops flexibly.

## Results

### Differently over-expressing miR398 promotes rice growth distinctly under normal conditions

**Figure 1:**
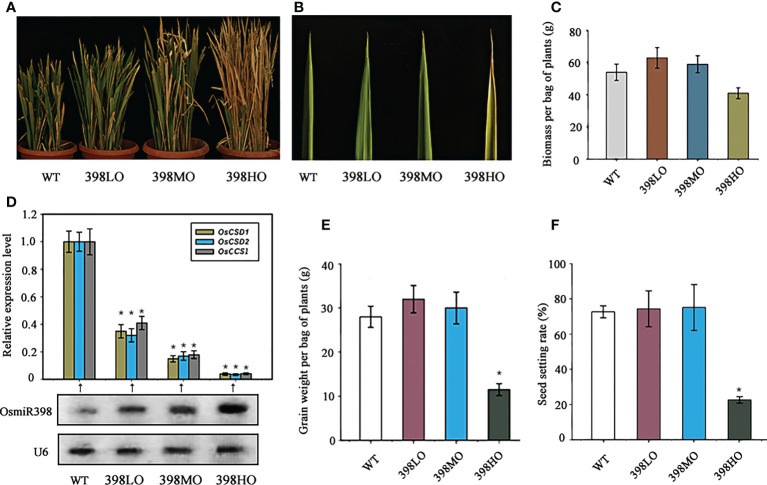
The agronomic performances of different levels of over-expressions of miR398 in rice under normal conditions. **(A)**. Morphologies of the WT (wild-type), 398LO (miR398 low level of over-expression), 398MO (miR398 moderate level of over-expression), and 398HO (miR398 high level of over-expression) at the mature stage. **(B)**. Phenotypes of the upmost leaf of the WT, 398LO, 398MO, and 398HO lines at the elongating stage (90d-old). **(C)**. Biomass (dry weight except grains) per bag of plants of the WT, 398LO, 398MO, and 398HO at the mature stage. **(D)**. Relative expression level of miR398 and its three targets in the WT, 398LO, 398MO, and 398HO lines. Targets were measured by Quantitative Reverse Transcription-PCR (qRT-PCR) and miR398 was detected by northern blot. U6 served as loading control. **(E)**. Grain weight per bag of plants of the WT, 398LO, 398MO, and 398HO after harvest. **(F)**. Seed setting rates of the WT, 398LO, 398MO, and 398HO lines at the mature stage. Asterisks indicate significant difference at P ≤ 0.05 compared with the wild-type by Student’s *t* test (n = 3; means ± SD).

Plant *SODs* are induced by various stresses, indicating their roles in the stress-response (Sharma and Davis, 1997; Kliebenstein et al., 1998). the products of stress-related genes usually not only boost plant abilities of resisting stresses but also have negative feedback on cell division and expansion, and thus result in growth inhibition (Liu et al., 1998; Zhu, 2001). Logically, different degrees of suppression of *SODs* may release this growth inhibition and prompt plant growth distinctively. microRNAs, as a class of sensitive regulators, would help to achieve this goal because three stress-related genes, *OsCSD1* (*Os03g22810*), *OsCSD2* (*Os07g46990*), and a *Copper Chaperone for Cu/Zn Superoxide Dismutase1* (*OsCCS1*, *Os04g48410*), are targeted by miR398 in rice (Li et al., 2010; Lu et al., 2011; Zhang et al., 2017). Here we separately overexpressed miR398 under three promoters, *Ubiquitin 1* (*pUbi l*), *CaMV35S* (*Cauliflower Mosaic Virus*) and an attenuated *CaMV35S* in rice subspecies *“Ningjing 8”*, which is widely planted in the southeast of China. *Ubiquitin 1* promoter is a powerful constitutive promoter that has been proven to have much stronger activities than *CaMV35S* promoter in the monocotyledons (Christensen et al., 1992; Muhitch and Shatters, 1998). Attenuated *CaMV35S* promoter was generated by deducting a sequence of enhancer from the original *CaMV35S* promoter. Then the three transgenic lines were selected on *hygromycin* in T_1_ generation and transferred into barrels filled with fertile soil. All three transgenic lines and the wild-type were grown under natural long-day conditions with a regular supply of nutrition and water. Their agronomic traits were investigated in detail all the life time. 2-week-old seedlings were chosen for gene expression analysis performed by northern blot for measuring small RNA and by quantitative Reverse-Transcription-PCR (qRT-PCR) for detecting mRNA. Northern blot showed miR398 was overexpressed in all three transgenic lines with different levels (**Figure 1D**). Compared with the wild-type, miR398 was overexpressed in the transgenic lines roughly at high, moderate, and low levels separately driven by *pUbil1*, *p35S*, and attenuated *p35S*. We named the three transgenic lines 398HO (miR398 high level of over-expression), 398MO (miR398 moderate level of over-expression), and 398LO (miR398 low level of overexpressing). Meanwhile, the expressions of the three targets were exhibited in roughly reverse strength in the three lines (**Figure 1D**). The growth rates of the three transgenic lines were almost identical to the wild-type in the seedling stage but differences gradually emerged in the elongation stage: 39HO grew fastest, 398HO secondly, 398LO thirdly, and the wild-type slowest. Ultimately, the lengths of the transgenic lines in the adult stage fit the tendency of the expression levels of miR398, namely, the 39HO lines were the tallest, followed by the 398HO, the398LO, and the wild-type (**Figure 1A**). Although 398HO lines grew fastest, they also exhibited some other symptoms such as rust leaves and withered stems (**Figure 1A, B**). The biomass (dry weight except grains) of the 398LO line had the biggest weight, with 398MO exhibiting the second biggest, however, the biomass of 398HO was much lower than the wild-type (**Figure 1C**). Similarly, the grain weights of the three lines tallied with the tendency of their biomass performances and that of 398HO was significantly reduced (**Figure 1E**). The grain weight per bag of plants of the wild-type was 28g while that of 398LO and 398MO was 32g and 30g respectively, but 398HO was only 11.5g (**Figure 1E**). The yield was increased by 14.5% in the 398LO and by 7.3% in the 398MO. Per bag plants here means an independent line with an identical genetic background, including all tillers grown up from the same one seed. In addition, 398LO and 398MO also displayed slightly higher levels of seed setting rate than the wild-type, while that of 398HO showed a significantly lower level (**Figure 1F**). These data suggested that reducing the expression of rice *OsCSD1-2* and *OsCCS1* genes at a mild degree through over-expressing miR398 could be an effective way to promote rice growth and increase grain yield. However, excessively reducing their expression would cause vein growth which in turn causes the loss of both the biomass and grain yield.

### Lines overexpressing miR398 are susceptible to saline soil

**Figure 2:**
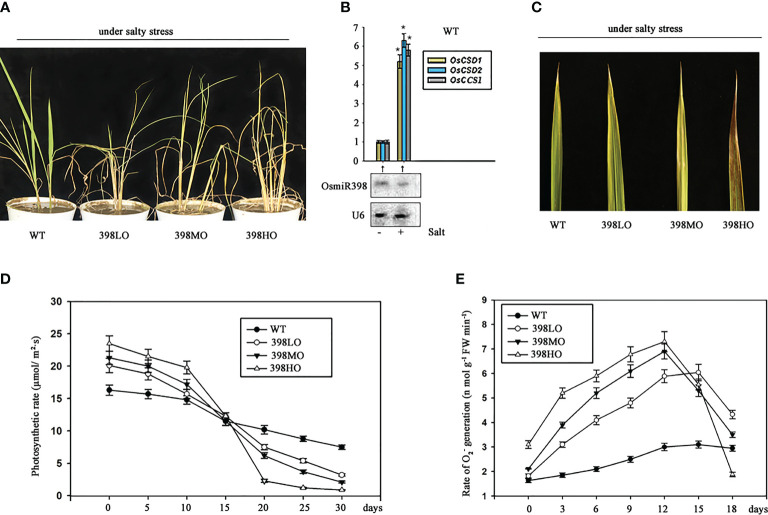
The agronomic performances of different levels of over-expressions of miR398 in rice under salt stress. **(A)**. Morphologies of the three transgenic lines overexpressing miR398 under mild salt stress after one month of treatment. **(B)**. The responses of miR398 and its three targets to salt stress in the wild type. The expression of miR398 was detected by northern blot and the expressions of its targets were measured by qRT-PCR after two days of treatment. “-” means no salty stress and “+” means imposed salt stress. U6 served as loading control for small RNA. Asterisks indicate significant difference at P ≤ 0.05 compared with the no salt stress by Student’s *t* test (n = 3; means ± SD). **(C)**. Phenotypes of the upmost leaf of the WT, 398LO, 398MO, and 398HO lines after 3 weeks of treatment. **(D)**. Time course of photosynthetic rate in the wild-type, 398LO, 398MO, and 398HO lines under salt stress. The photosynthetic rates were measured by detecting CO_2_ exchanging rate after being transplanted into salty soil. **(E)**. Changes of cellular O_2_^–^ generation rates in the wild-type, 398LO, 398MO, and 398HO lines under salt stress. All data was the means of three independent measurements ± s.e. in Figure D and E.

398LO and 398MO were able to increase biomass and grain yield under normal conditions in pots (**Figure 1C, E**), but it was unknown whether they also perform well under salt situation, a common stress in agriculture. Here we cultivated the three transgenic lines overexpressing miR398 under a real saline soil transported from a typical salt land spot in Binghai County, which is close to the coast of the east China sea, Jiangsu province, China. It has been known the salt condition of this region can represent a large area of saline land in eastern Asian. The soil samples were taken from 16 random sites distributed in different fields of that county. If the extracted solution of soil has an electrical conductivity of 20 mM or more then the soil is considered as saline or salt-affected soil (de Souza Silva and Fay, 2012; Numan et al., 2018). The electrical conductivity values of all these samples approached ~ 42.15 mM without significant fluctuations. Then all the soil samples were mixed together. One-week-old seedlings of all three transgenic lines and the wild-type growing under normal conditions were transplanted into the barrels filled with mixed saline soil for further cultivating. They all grew under long-day conditions with a regular supply of nutrition and water. The seedlings of 398HO began turning yellow only 5 days after being transplanted. 2 weeks later, when the wild-type lines start becoming yellow, the other three overexpressing lines showed more yellow and withered symptoms. After one month of treatment, the leaves of all three genetic lines presented severe dehydration symptoms (**Figure 2A**). Roughly, the severities of the withered symptom positively fit with the degrees of over-expression of miR398 (**Figure 2C**). The wild-type plants could barely survive in the salty soil with a few seeds, but the other three lines were not able to live for 60 days and 398HO lines died earliest at the 40th day of being transplanted. Although the growth rates of the three transgenic lines were higher than that of the wild-type plants at the first week, their growth rates slowed down two weeks later. The photosynthetic rates of the transplants lines become slower on the 15th day after being transplanted, even though they had slightly higher rates at the earlier stage (**Figure 2D**). It is well known that upregulated *SODs* were caused by downregulated miR398 under various stresses (Sunkar et al., 2006; Lu et al., 2011). The expression of miR398 was down-regulated and the expressions of its three targets were up-regulated in the wild-type under salt stress (**Figure 2B**). SODs are known as a class of scavenger of oxygen free radicals under stresses. We found that the generating rates of O_2_^–^ increased during the first 12 days and then dropped down sharply in the three transgenic lines growing in salty soil (**Figure 2E**). The increased level of O_2_^–^ might be caused by suppressed SODs (**Figure 1D**) but the sharp drop of O_2_^–^ likely resulted from the declined physiologic activities of the transgenic lines living under salty stress for too long. These results indicated that the transgenic lines overexpressing miR398 are susceptible to salty soil.

### Growth of miR398 short tandem target mimic lines is suppressed under normal conditions

**Figure 3:**
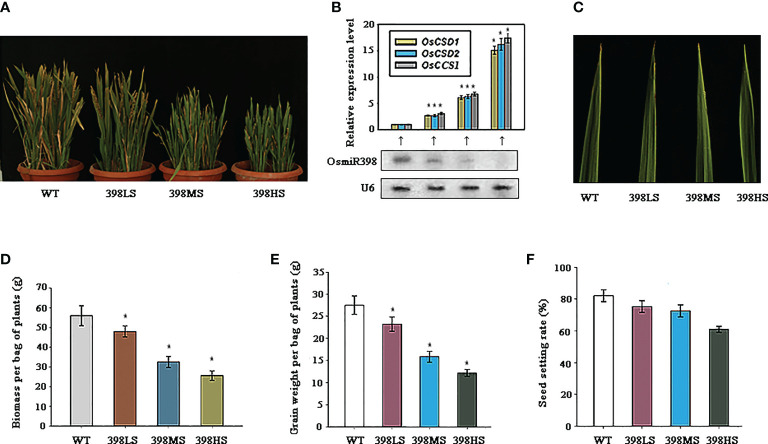
The growth traits of miR398 STTM (short tandem target mimic) lines under normal conditions. **(A)**. Gross morphologies of the WT, 398LS (miR398 low level of STTM), 398MS (miR398 moderate level of STTM), and 398HS (miR398 high level of STTM) at the mature stage. **(B)**. Expression levels of miR398 and its three targets in the WT, 398LS, 398MS, and 398HS lines. Targets were measured by quantitative Reverse Transcription-PCR (qRT-PCR) and miR398 was detected by northern blot. U6 served as loading control. **(C)**. Phenotypes of the upmost leaves of the WT, 398LS, 398MS, and 398HS at the elongating stage (90d-old). **(D)**. Biomass (dry weight) per bag of plant of the WT, 398LS, 398MS, and 398HS at the mature stage. **(E)**. Grain weight per bag of plants of the WT, 398LS, 398MS, and 398HS after harvest. **(F)**. Seed setting rates of the WT, 398LO, 398MO, and 398HO at the mature stage. Asterisks indicate significant difference at P ≤ 0.05 compared with the wild-type by Student’s *t* test (n = 3; means ± SD).

Technology of short tandem target mimics (STTMs) is an effective tool to inhibit the expression of a given microRNA (Tang et al., 2012; Yan et al., 2012). An earlier strategy for a given target to escape the control of a microRNA is to mutate the sites directed by microRNA in the target mRNA without alteration of amino acids (Jones-Rhoades et al., 2006). But this strategy makes it difficult for all targets to escape from control of that microRNA. STTM technology can facilitate the aim to escape all targets from the regulation of that microRNA and the efficiencies depend on the degree of inhibition of the microRNA (Teotia et al., 2017; Wang et al., 2019). Here we constructed the STTM vector of miR398 and drove its expression under promoters of *Ubiquitin 1*, *CaMV35S*, and attenuated *CaMV35S*, respectively. Many independent transgenic lines were generated and planted in barrels along with the wild-type. Molecular detection showed that the expressions of miR398 were inhibited in all three lines, 398LS (miR398 low degree of STTM), 398MS (miR398 moderate degree of STTM), and 398HS (miR398 high degree of STTM), and the degrees of inhibitions were in line with the strength of the promoter (**Figure 3B**). By contrast, the expressions of the three targets were enhanced significantly and were in reverse trends with miR398 (**Figure 3B**). The growth of the three lines was also suppressed all the time and their leaves were a darker green than the wild-type (**Figure 3C**). At the mature stage, partial leaves of the wild-type turned yellow but the three STTM lines still remained green and 398HS kept the greenest and little dwarf phenotype (**Figure 3A**). The suppression of growth in the STTM lines was further verified by biomass analysis after full maturation. As **Figure 3D** shows, the weights of the biomass of all three lines were lower than the wild-type and that of 398HS was the lowest one, about half that of the control (**Figure 3D**). All lines were able to produce seeds and the yields of the three STTM lines were reduced. The weight of grain of the wild-type per bag (including all tillers) was 27.5g on average, while the three STTM lines were 23.2g (398LS), 19.8g (398MS), and 16.4g (398HS) respectively (**Figure 3E**). However, the statistics of the seed setting rate showed that, of the three, STTM lines were slightly lower than that of the wild-type (**Figure 3F**). These results suggest that the growth rate, especially some important agronomic traits such as biomass, grain weight, and seed setting rate, are suppressed in the miR398 STTM lines.

### miR398 STTM lines can tolerate stress in salty soil

**Figure 4:**
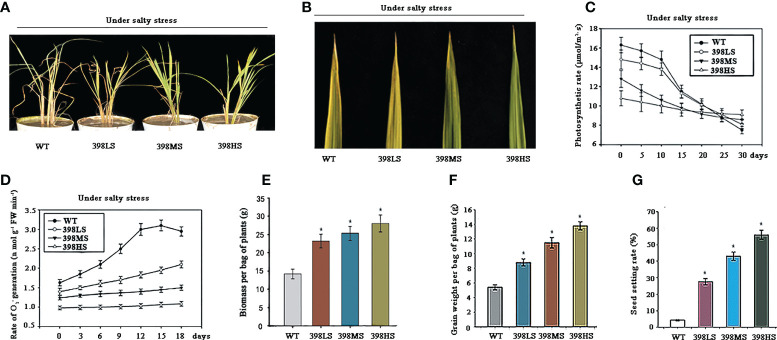
The growth traits of the three miR398 STTM lines under salt stress. **(A)**. The phenotypes of the three miR398 STTM lines under salt stress after one month of treatment. **(B)**. Phenotypes of the upmost leaf of the WT, 398LO, 398MO, and 398HO lines at the elongation stage. **(C)**. Time course of photosynthetic rate in the WT, 398LO, 398MO, 398HO under salt stress. The photosynthetic rates were measured by detecting CO_2_ exchanging rate after being transplanted into salty soil. **(D)**. Changes of cellular O_2_^–^ generation rates in the wild-type, 398LO, 398MO, and 398HO under salt stress. All data were the means of three independent measurements ± s.e. in Figure C and D **(E)**. Biomass per bag of plants of the WT, 398LS, 398MS, and 398HS at the mature stage. **(F)**. Grain weight per bag of plants of the wild-type, 398LS, 398MS, and 398HS after harvest. **(G)**. Seed setting rates of the WT, 398LO, 398MO, and 398HO at the mature stage. Asterisks indicate significant difference at P ≤ 0.05 compared with the wild-type by Student’s t test (n = 3; means ± SD) in the Figure E, F, and G.

Based on the known roles of the targets of miR398 and their up-regulated expression level in the three STTM lines, we speculated whether they could resist the stresses under salty soil. One week-old-seedlings of the wild type and the three miR398 STTM lines which were germinated under normal condition were transplanted into barrels containing the same mixed salty soil as described previously. Two weeks later, when the leaves of the wild-type turned yellow, the other three STTM lines remained green. One month later, the three STTM lines presented better appearances than the wild type and the 398HS line was the best one(**Figure 4A**). All plants could survive in the saline soil and the three STTM lines displayed much better agronomic performances. At the elongation stage, the upmost leaves of the 398HS and 398MS lines were still able to remain green but that of the wild type and 398LS had already turned quite yellow (**Figure 4B**). The leaves of the same position of the 398LS had an approximate phenotype to the wild type but with a slightly inferior symptom (**Figure 4B**). The photosynthetic rates in all plants were measured before and after being transplanted into saline soil. **Figure 4C** shows that the photosynthetic rate of the wild-type was higher than all STTM lines under normal conditions and even after two weeks of treatment with salt. However, the photosynthetic rate of the 398HS lines can maintain a nearly constant level while the other lines’ levels, especially the wild-type and 398LS, dropped down after 30 days of treatment. A month later, surprisingly, the photosynthetic rate of 398HS maintained the highest level even though it was at the lowest level at the beginning (**Figure 4C**). As **Figure 3B** shows, the expression levels of the *OsCSD1*, *OsCSD2*, and *CCS1*are differently up-regulated in the three STTM lines, so we speculated whether the increased level of the *CSDs* help to scavenge oxygen free radicals. The O_2_^–^ levels were measured since all STTM lines and the wild-type were transplanted into saline soil. As **Figure 4D** shows, the O_2_^–^ level of the wild type is higher than that of the three STTM lines and that of the 398HS lines was the lowest one. The O_2_^–^ level gradually increased in the wild-type and slightly rose in the 398LS line under saline conditions, while that of 398MS and 398HS lines almost remained constant, indicating the increased level of *OsCSDs* helps to scavenge active oxygen. These results suggest that the STTM lines of miR398 can boost plant ability to survive in saline stress. As **Figure 3A** shows, the growth of all the STTM lines was suppressed under normal conditions, but we did not know whether this phenomenon could reappear under saline conditions. All tested lines were able to survive in the mixed soil with a mild degree of salt stress, but the three STTM lines had a significant increase of biomass over the wild-type (**Figure 4E**). Markedly, the three miR398 STTM lines also had a significant increase of grain weight, 9.1g of 398LS, 11.5g of 398MS, and 14.6g of 398HS, while the grain yield of the wild-type was very low, just 5.2g (**Figure 4F**). Statistics of seed setting rates also showed that the three miR398 STTM lines are significantly higher than that of the wild-type (**Figure 4G**). These results suggested that, compared with the wild-type, miR398 STTM lines can boost their agronomic performances under salty soil.

## Discussion

Salinity is a major concern in modern agriculture due to frequent irrigation, industrial pollutants, sea water erosion, and climate change (Ganie et al., 2019). It is estimated that high salinity reduced agricultural yields by more than 20% in cultivated lands worldwide (Ganie et al., 2019). The human population is expected to reach 10 billion by the middle of the century, which demands a 70-110% increase in crop productivity (Tilman et al., 2011). This goal is expected to be accomplished in both salty and arable land. However, the deteriorating situations make this goal a bigger challenge. Originally, scientists attempted to gain salt-resistant varieties *via* conventional breeding, but unfortunately, few suitable genetic model systems have been found. Moreover, crop-closed halophytes that can survive in the salty soil usually have much lower grain yields (van Zelm et al., 2020). With the development of molecular cloning, gene engineering is another possible alternative for modifying crop’s traits to better survive in both salty and normal soil (Deinlein et al., 2014; Munns and Gilliham, 2015). Our study here shows that flexible manipulation of a regulator, miR398, can increase rice yield in accordance with specific soil conditions. Limited by the tested number of samples under conditions of greenhouse, more data from field trials is needed to further confirm the conclusions reached by this study.

As a well-known regulator, the function of miR398 depends on what its targets are and how it regulates the targets. The targets of miR398 have been identified as *Cu/Zn Superoxide Dismutases1-2*, *CCS* (copper chaperone of CSD), and *COX5b–1* in plants. Even though the expression of *CSDs* has been proven to be stress-induced in plants and was deemed as strong player in plant defense to various stresses (Sharma and Davis, 1997; Kliebenstein et al., 1998), it is also a fact that the growth of plants is suppressed under various stresses. It remains unclear whether the suppression of growth was caused by these stress-responsive genes. Earlier studies with transgenic overexpression of stress tolerance genes have also implied that there are relations between stress and growth regulation, for example, the constitutive expression of some stress-related genes such as *CBF1*(*C-repeat/DRE Binding Factor1*) and *ATHB7* (HD-Zip gene) has been shown to cause slow growth of transgenic plants (Söderman et al., 1996; Liu et al., 1998). So it may be a common mechanism for most stress tolerance genes to negatively regulate plant growth in response to various stresses. Their gene products or downstream target molecules probably actively feed into cell division and expansion machinery to result in growth inhibition and therefore they might represent ‘stress signals’.

As for *Cu/Zn Superoxide Dismutases1-2*, *CCS* (copper chaperone of CSD), and *COX5b–1* in plants, the functions of them would be beyond the scopes of current understanding. In addition to the *CSD* genes known as being involved in scavenging superoxide anions, the roles of *COX5b–1* (*subunit 5b of cytochrome c oxidase*) are believed to participate in the mitochondrial electron transport chain which drives oxidative phosphorylation (for production of ATP), suggesting the diverse functions of targets for miR398 to regulate. Here, we showed that upregulated expression of miR398 targets can inhibit the growth of rice under normal conditions but confers rice the ability to resist salt stress and thus relatively increase rice yield in salty soil. Meanwhile, downregulated expression of miR398 targets can release the inhibition of them upon rice growth and thus improve rice yield under normal conditions. Our results here were partially consistent with the report that changes in the expressions of miR398 could have an effect on rice yields (Zhang et al., 2017). Our results here would provide a practical strategy for molecular breeding by flexibly engineering a stress-related microRNA to optimize rice yields under different conditions.

## Methods

### Plant materials and growth conditions

Rice cultivar subspecies (*Oryza sativa* ssp. *Ningjing 8*), which is widely planted in the southeast of China, was used as the control plants and served as the genetic background for all tested plants. All seeds involved in this study were taken from the Key Laboratory of Plant Functional Genomics of the Ministry of Education, Yangzhou University, China. Y.K. undertook the formal identification of the plant materials used in his study. The conditions for normal growth of the control plants and transgenic plants were performed as described by Lu et al. (2020). The tested seeds were germinated in petri dishes (diameter, 90 mm) containing MS liquid medium for one week and then were transplanted into pots or barrels containing fertile soil transported from our laboratory field. To simulate salty conditions, the excavated soil samples from 16 spots in Binghai County, eastern region of Jiangsu province, China, were mixed together after every one’s electrical conductivity was measured; then the one-week-old seedlings of the wild-type and transgenic lines were transplanted into pots or barrels filled with mixed soil. Water was irrigated regularly.

### Measuring expressions of genes

For quantitative RT-PCR analysis of *OsCSD1* (*Os03g22810*), *OsCSD2* (*Os07g46990*), and a *Copper Chaperone for Cu/Zn Superoxide Dismutase* (*CCS1*, *Os04g48410*), 2 μg of total RNA was reversely transcribed in a total volume of 20 μL with 0.5 mg oligo (dT)15, 0.75 mM dNTPs, 10 mM DTT, and 100 U SuperScript II RNase H2 reverse transcriptase (Invitrogen). The reaction volume for PCR was 20 μL with 1 μL of the RT reactions (Lu et al., 2018). The primers for quantitative RT-PCR are listed as the following: *OsCSD1*, FW: 5’- CTTGGAAATGTCACCGCTGGA -3’, RV: 5’- CTTGAAGTCCGATGATCCCGC -3’; *OsCSD2*: FW: 5’- ACTGGAGCACACTCCATCATTGG -3’, RV: 5’- CTAACCCTGGAGTCCGATGATTC -3’, *CCS1*: FW: 5’- GATTGGCCGCTCTATTGCGTTG -3’, RV: 5’- TGACTCCCAAATGGTGACACC -3’; 30 cycles for PCR was performed and the expression levels of the samples were normalized by *OsUbiquitin* gene (Forward: 5’-AACCAGCTGAGGCCCAAGA-3’, Reverse: 5’-AACCAGTCCATGAACCCGG-3’). Experiments were performed with three biological replicates, of which each was performed in three technical replicates. To survey the expression level of miR398, total RNA was extracted from different tissues by using TRIzol reagent (Invitrogen). The DNA oligonucleotides of 5’-AAGGGGTGACCTGAGAACACA-3’ served as probe for miR398, 5’-ATTTCTCGATTTGTGCGTGTC-3’ for U6. The two probes were labeled with γ-^32^P-ATP at 5′ terminus. The process was performed as described previously (Lu et al., 2018).

### Construction of expression vector and generation of transgenic rice lines

To highly overexpress OsmiR398, a fragment of genomic DNA with a length of 165bp containing the flank sequence of OsmiR398a was amplified by using the primers (FW: 5’-GC GAGCTC ATTGGACAAAGAATCATGAGAA-3’; RV: 5’-AA GGATCC AGCAATGAAGAGAAGCTGAACC-3’; BamH I and *Sac I* sites are underlined respectively). Then the fragment was inserted into expression vector, pCAMBIA 1301, driven by the original promoter of *Ubiquitin 1*. To moderately and lowly overexpress OsmiR398, we cloned the full length of promoter of *CaMV35S*, whose length is 538 bp, with primers (FW: GG AAGCTT catggagtcaaagattcaaatag; RV: AA GGATCC AGTCCCCCGTGTTCTCTCCAAATG) and replaced original *Ubi1* promoter by 35S promoter (*Hind* and *BamH I* sites are used). To generate the attenuated *CaMV35S* promoter, the trunked fragment of *CaMV35S* promoter of 342 bp was cloned using primers (FW: GG AAGCTT caaagggtaatatccggaaacc; RV: AA GGATCC AGTCCCCCGTGTTCTCTCCAAATG. *Hind* and *BamH I* sites are used) to replace the original *Ubiquitin 1* promoter. Then the precursor of miR398a was inserted under full and partial length of promoter of *CaMV35S* separately.

To generate the different STTM lines of miR398, the STTM structure was designed as the following sequence: 5’AAAAGGGGTGACCctaTGAGAACACAgttgttgttgttatggtctaATTTAAATatggtctaaagaagaagaatAAGGGGTGACCctaTGAGAACACACG 3’ (Tang et al., 2012). Then the three kinds of pCAMBIA 1301 vectors containing *Ubiquitin 1*, *CaMV35S*, and attenuated *CaMV35S* promoter linked with designed STTM structure were synthesized respectively by GENEWIZ biotech Company, SuZhou city, China. All the constructed expression vectors were introduced into rice *calli* through *Agrobacterium tumefaciens* (EHA105) mediated methods (Hiei et al., 1994).

### Testing photosynthetic rate

An average of three plants from all transgenic lines and the wild type were used to test photosynthetic rate using Li-6400 photosynthetic instrument. The upmost leaves were chosen to determine the photosynthetic rate. The light intensity in the leaf chamber was controlled at a range of about 800 μmol·(m^2^·s)^-1^. The size of the leaf chamber was 2 cm × 3 cm and temperature was controlled at 25 °C, with three repeats for every test.

### Quantitative assay of cellular O_2_^–^ generation

The method for detection of O_2_^-^ generation rate was described by Lu et al., 2011. 0.5 g fresh leaves were ground in 65 mM PBS (pH7.8) and then centrifuged at 5000*g* for 10 min. 1 mL suspension was taken and mixed with 0.1 mL hydroxylamine chloride (10 mM). After 20 min of reaction at room temperature, 1 mL sulfanilic acid (17 mM) and 1 mL a-Naphthylamine (7 mM) were added to the above reaction solution for another 20 min. The reaction mixture was then extracted with ethyl ether. The aqueous phase was taken and its optical absorbance was measured at 530 nm. Three measurements were conducted for each treatment.

## Data availability statement

The original contributions presented in the study are included in the article/supplementary material. Further inquiries can be directed to the corresponding authors.

## Author contributions

YL performed most gene expression work. KY and YM took charge of cloning genes as well as constructing vectors. YZ completed most work on transforming rice. ZG investigated all the agronomic traits. YL and QL analyzed data and wrote the article. All authors contributed to the article and approved the submitted version.

## Funding

This work was supported by the ‘National Natural Science Foundation of China (No. 31271623)’ and the open fund of ‘Key Laboratory of Plant Functional Genomics of the Ministry of Education (No. ML202004)’. The funder did not play any roles in the design, analysis, interpretation of this study or relevant data.

## Acknowledgments

We thank Dr. Xiaofeng Cui for his critical comments and helpful discussion on this manuscript.

## Conflict of interest

The authors declare that the research was conducted in the absence of any commercial or financial relationships that could be construed as a potential conflict of interest.

## Publisher’s note

All claims expressed in this article are solely those of the authors and do not necessarily represent those of their affiliated organizations, or those of the publisher, the editors and the reviewers. Any product that may be evaluated in this article, or claim that may be made by its manufacturer, is not guaranteed or endorsed by the publisher.
